# Comparison of morphological changes in efferent lymph nodes after implantation of resorbable and non-resorbable implants in rabbits

**DOI:** 10.1186/1475-925X-10-32

**Published:** 2011-04-26

**Authors:** Alexandr Bondarenko, Marion Hewicker-Trautwein, Nina Erdmann, Nina Angrisani, Janin Reifenrath, Andrea Meyer-Lindenberg

**Affiliations:** 1 Small Animal Clinic, University of Veterinary Medicine Hannover, Bünteweg 9, 30559 Hannover, Germany; 2 Departement of Pathology, University of Veterinary Medicine Hannover, Bünteweg 17, 30559 Hannover, Germany; 3 Department of Pathology, Dnipropetrovs'k State Medical Academy, str. Zhovtneva ploshcha 14, 49005 Dnipropetrovs'k, Ukraine; 4 Clinic for Small Animal Surgery and Reproduction, Centre of Clinical Veterinary Medicine, Faculty of Veterinary Medicine Ludwig-Maximilians-Universität München, Veterinärstr. 13, 80539 München, Germany

## Abstract

**Background:**

Magnesium alloys as biodegradable implant materials received much interest in recent years. It is known that products of implant degradation can induce several types of immune response. Hence, the aim of this study was to examine the morphological changes of efferent lymph nodes after implantation of different resorbable magnesium alloys (MgCa0.8, LAE442) in comparison to commercially available resorbable (PLA) and non-resorbable (titanium) implant materials as well as control groups without implant material.

**Methods:**

The different implant materials were inserted intramedullary into the rabbit tibia. After postoperative observation periods of three and six months, popliteal lymph nodes were examined histologically and immunhistologically and compared to lymph nodes of sham operated animals and animals without surgery. Haematoxylin and eosin staining was performed for cell differentiation. Mouse anti-CD79α and rat anti-CD3 monoclonal primary antibodies were used for B- and T-lymphocyte detection, mouse anti-CD68 primary antibodies for macrophage detection. Evaluation of all sections was performed applying a semi quantitative score.

**Results:**

The histological evaluation demonstrated low and moderate levels of morphological changes for both magnesium alloys (LAE442 and MgCa0.8). Higher than moderate values were reached for titanium in sinus histiocytosis and histiocytic apoptosis (3 months) and for PLA in histiocytic apoptosis (3 and 6 months). The immune response to all investigated implants had a non-specific character and predominantly was a foreign-body reaction. LAE442 provoked the lowest changes which might be due to a lower degradation rate in comparison to MgCa0.8. Therewith it is a promising candidate for implants with low immunogenic potential.

**Conclusion:**

Both examined magnesium alloys did not cause significantly increased morphological changes in efferent lymph nodes in comparison to the widely used implant materials titanium and PLA. LAE442 induced even lower immunological reactions. Therewith MgCa0.8 and especially LAE442 are appropriate candidates for biomedical use.

## Background

Nowadays there are many studies dedicated to the research of biodegradable implants' influence on living tissues [1-8]. An important aim of these studies is the investigation of immunological effects of biodegradable materials [9-18].

Degradation products of metallic biomaterials include particulate wear debris, colloidal organometallic complexes (specifically or non-specifically bound), free metallic ions, inorganic metal salts or oxides and precipitated organometallic storage forms [9]. If the different substances with a various biochemical activity are present in a local area, the metal implants may influence the immune system in different possible ways: immune response mediated by type IV delayed hypersensitivity (DTH) [9,10,17,18], immune suppression via apoptosis of responsible cells [19-22] and foreign-body reaction [5].

Even in spite of chemical inertness of metals like titanium, corrosion processes in contact with biological systems (aging of prosthesis) are described [23,24], accompanied by release of ions, which are not sensitizers on their own, but can induce the immune system by generating complexes with native proteins [10-14,25-27]. These metal-protein complexes are supposed to be candidate antigens (i.e. allergens) for developing delayed hypersensitivity [11]. DTH based on interactions between antigen-presenting cells, which process and present antigen, and CD4+ T-cells, which initiate this type of immune response by the release of cytokines and by macrophage activation [9].

Candidate antigen-presenting cells in the periimplant region include macrophages, endothelial cells, Langerhans cells, dendritic cells and to lesser extent parenchymal tissue cells [11]. The clonal T-lymphocyte specificity associated with type IV delayed hypersensitivity remains the dominant mechanism associated with implant related hypersensitivity responses [11-13].

Resorbable implant materials, inclusive magnesium containing alloys, have been investigated as sources of hypersensitivity-type immune responses. Witte et al. [3] demonstrated the absence of skin sensitizing properties for standard implant materials PLA (SR-PLA96) and titanium (TiAl6V4) as well as for the investigated magnesium alloys (AZ31, AZ91, WE43, and LAE442). However, immunogenic reactions associated with polymers have been reported, albeit less frequently [15]. Additionally, the sensitizing properties of different metals (haptenic components in antigens) are described [16]. Also, occasional responses to titanium have been demonstrated [17,18].

The induction of apoptosis, that can reflect the immunosuppressive activity of implants, was reported for magnesium and calcium [28-30], rare earth elements [28], titanium particles [19,20,23] and PLA [22].

Inflammatory host responses to different resorbable implant materials in local area have been well described in several studies [1,4,5]. Implantation of PLA as well as magnesium-containing resorbable materials induced the insignificant non-specific inflammation in surrounding tissues and demonstrated a moderate level of neutrophilic and macrophage infiltration, presence of giant foreign-body cells and small amount of T-lymphocytes with a decrease tendency of these parameters from three to six months of implantation period [4,5]. Unfortunately, just a few of contemporary studies described morphological changes in the regional lymph nodes after the intraosseous implantation of PLA [31] and in spleen after the drainage of different metal ions [32], though it is well known that any inflammatory stimuli involve the lymph nodes, which act as defensive barriers [33]. Thus, any immune response against foreign antigens is often associated with lymph node enlargement (lymphadenopathy) [33]. This condition can assume one of three patterns, depending on the causative agent: follicular hyperplasia, which is associated with B-cell activation, paracortical hyperplasia that shows reactive changes within the T-cell regions or sinus histiocytosis [33]. Additionally, some researchers reported the cases of sinus histiocytosis in regional lymph nodes induced by the implantation of prostheses contained chromium, cobalt and titanium [34,35].

Hence, in consideration of current data, an evaluation which could reflect the real condition of local and systemic immunity in patients with biodegradable implants and which would allow evaluating the host reaction is missing. Moreover, the appearance of such new biodegradable materials as magnesium-calcium alloys or magnesium-rare earth metal alloys [1,36-38] combined with deficiency of data about their immunogenic properties determines the actuality and necessity of the study of morphological changes in lymphoid tissue under the influence of these materials.

The aim of this study was to compare changes in popliteal lymph nodes after tibial bone implantation of magnesium alloys with standard implant materials in a rabbit model using histological and immunohistochemical techniques.

## Methods

For this study, the popliteal lymph nodes as regional drainage collectors of animals with the implanted magnesium-based alloys MgCa0.8 and LAE442 were examined in comparison to the established materials PLA (resorbable) and titanium (non-resorbable) as well as control groups without implant material.

### Implant material

The magnesium alloy MgCa0.8 contains adjacent to magnesium 0.8 wt% calcium, the alloy LAE442 the elements lithium (4 wt%), aluminium (4 wt%) and a rare earth composition metal (2 wt%). Main fractions in the composition metal were cerium (1.3 wt%), neodymium (0.37 wt%) and lanthanium (0.5 wt%). The alloy designation for LAE442 is in accordance with the ASTM-standard (American Society for Testing Materials). The alloy designation for MgCa0.8 is based on the glossary of chemical elements. The extruded magnesium implants of 25 mm length and 2.5 mm in diameter were produced according to previous studies [2,6]. Titanium implants (TiAl6V4, 25 mm length and 2.5 mm in diameter, Synthes, Germany) and PLA implants (poly-96L/4D-lactide, 25 mm length and 2.0 mm in diameter, Synthes, Germany) were commercially available.

### Animal Model

The animal experiment was authorized according to the German Animal Welfare Act and registered as number 509.6-42502/3-04/750. All materials were implanted intramedullary into both tibiae of adult, female New Zealand White rabbits (average weight 3.5 kg) with implantation periods of three and six months. Five rabbits were used for each group containing magnesium alloys, two rabbits were used for the PLA-group and two for the titanium-group.

As control groups, three rabbits in each time group received the same surgical procedure without pin implantation. An intramedullary trauma was caused with a plastic pestle (sham operated group) to differentiate a postoperative local inflammation from an implant induced inflammatory reaction. Six lymph nodes of animals which were used in other studies without surgery on the limb served as control for physiological lymph node morphology (control group).

Anaesthesia, surgical procedure and euthanasia as well as the examination of the bone-implant compound were described in detail by Krause et al. [2] and Thomann et al. [6].

### Histological and immunohistochemical setup

One popliteal lymph node of each group was explanted, fixed in 4% formalin (48 h, room temperature) and routinely embedded in paraffin.

Sections of 2-3 μm thickness were performed using a microtome (RM2255, Leica, Germany). Every explored lymph node was cut in 2-3 subsequent slices with interruption in 2-6 μm. For histological studies, the sections were stained with haematoxylin and eosin (H&E) and immunohistochemistry, which were performed according to established methods [39]. Antigen exposure was realised by heat-induced antigen retrieval (HIAR) method in citrate buffer (pH 6.0). Non-specific binding was reduced by incubation with normal goat serum (1:5) diluted in phosphate-buffered saline (PBS, pH 7.1) for 20 min at RT. Mouse anti-CD79α (HM 47/A9, Acris, Germany) for B-cell-detection, rat anti-CD3 (clone CD3-12, Serotec, Germany) for T-cell-detection and mouse anti-CD68 (EBM11, Dako, Germany) for the detection of histiocytes (macrophages) were used as the primary antibodies, which were diluted in PBS containing bovine serum albumin (BSA, 1%). The reactivity has been already tested on the rabbit animal model [39]. As secondary antibodies goat anti-mouse (biotinylated anti-mouse IgG (H+L), Vector Labs, Burlingame, CA) and goat anti-rat biotin-conjugated IgG (Biotinylated Anti-Rat IgG (H+L), Vector Labs, Burlingame, CA) was applied in concentration 1:200. Vectastain Elite ABC Kit (Biozol Diagnostica, Germany) was utilised as visualisation system. Diaminobenzidine (DAB) served as chromogen. The background was stained with Meyer's haemalaun. Negative control sections, in which the antibody was replaced by PBS, were included in all staining runs.

### Histological evaluation and scoring system

All sections were observed by light microscopy (Axio Imager Z1, Zeiss, Germany). The pictures were edited with Axio Vision imaging program (version 4.7.1).

**Figure 1:**
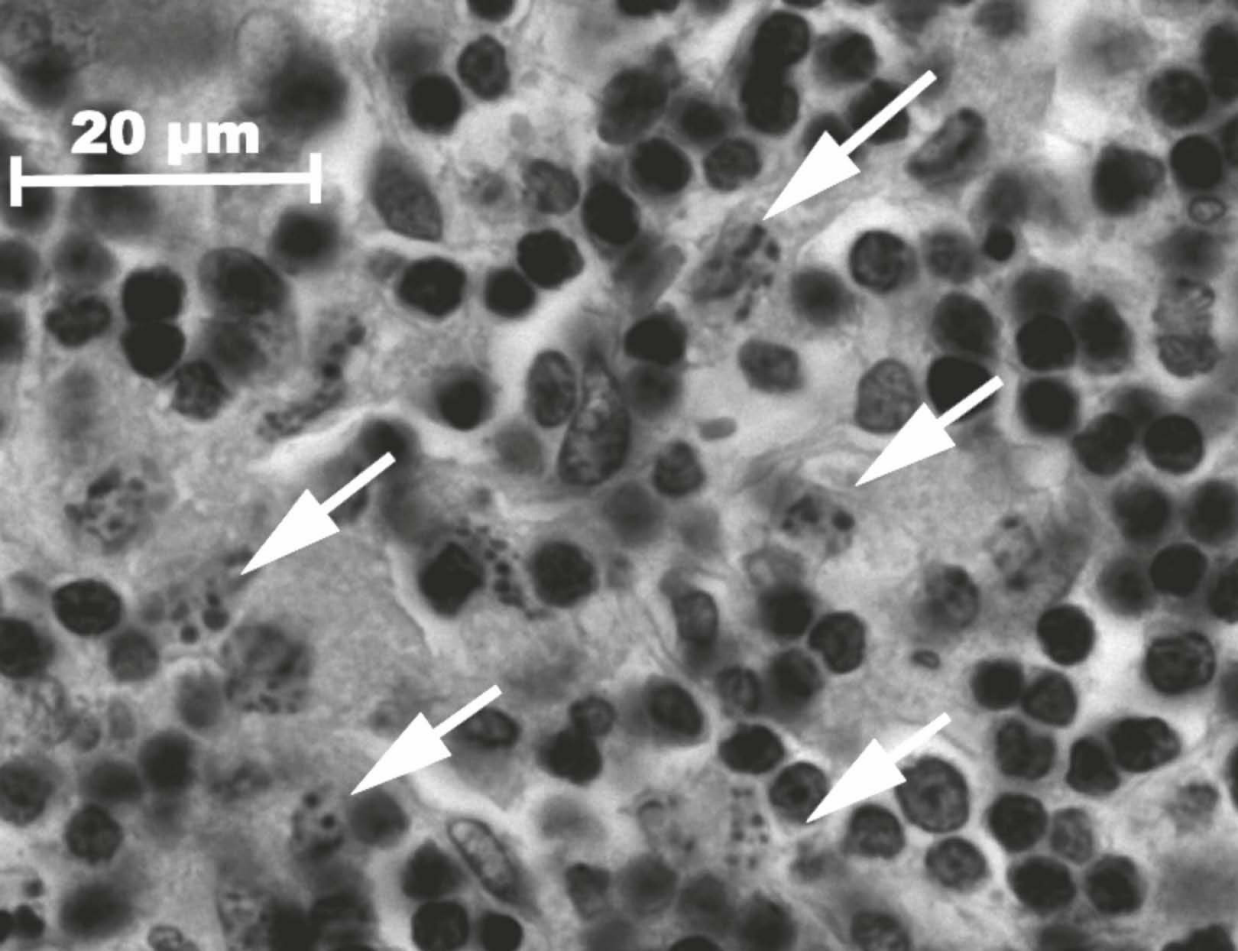
**Histiocytic karyorrhexis (arrows) as sign for apoptotic changes of histiocytes, (H&E, × 1000)**. Histiocytes with indicated apoptotic changes were counted and evaluated in accordance to the scoring system.

After a three times repeated evaluation of all sections, a specific set of the most abundant changes was established. Four of the most common characteristics (sinus histiocytosis, follicular hyperplasia, heterophilic infiltration and histiocytic apoptosis) were chosen. In H&E stained sections, heterophiles were counted. Sinus histiocytosis and follicular hyperplasia were evaluated semiquantitatively as a comparison of follicles and sinuses in lymph nodes of experimental groups with the size of appropriate compartments (follicles and sinuses respectively) of intact popliteal lymph nodes in the control group of rabbits without surgery (Table 1). The presence of B-cells, T-cells and histiocytes in H&E staining was confirmed by CD79α, CD3 and CD68 immunostaining. Apoptotic changes of histiocytes were detected in appearance of karyopyknosis, karyorrhexis (Figure 1) and, seldom, apoptotic bodies [21]. The histiocytes with indicated apoptotic changes were counted and evaluated in accordance to the scoring system (Table 1).

**Table 1:** Scoring system for the histological evaluation of different pathological changes in the lymph nodes

Score	Cell number per field of view		Level of compartment enlargement	
	heterophils	apoptotic histiocytes	sinus histiocytosis	follicular hyperplasia
0	0	0	-	-
1	1-15	1-5	+	+
2	15-25	5-10	++	++
3	26-35	11-15	+++	+++
4	36-45	16-20	++++	++++
5	> 45	> 20	+++++	+++++

### Statistics

The mean values of the semiquantitative scoring were tabulated and differences between the material and time groups statistically analysed using a Mann-Whitney U test (SPSS for Windows). P < 0.05 indicated statistical significance.

## Results

The most common morphological change in all investigated material groups was sinus histiocytosis, followed by heterophilic infiltration, appearance of histiocytic apoptosis, and rarely, occurrence of follicular hyperplasia. Distribution of these features in lymph nodes after exposure to different implant materials was not equally.

**Figure 2:**
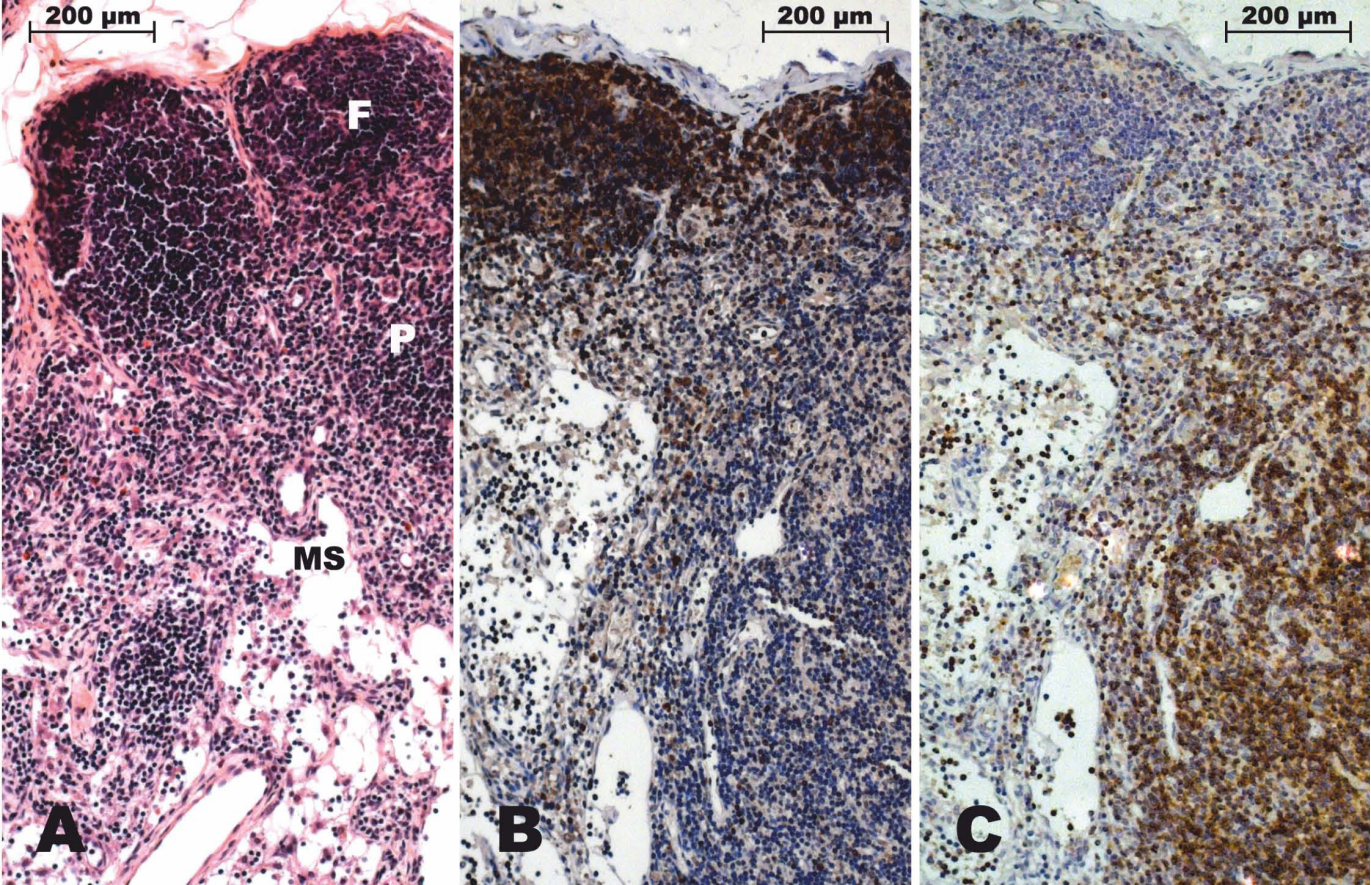
**Physiological structure of a lymph node from a rabbit of the control group without surgery**. **A**. Routine histological staining allows the recognition of different compartments: follicles (F), paracortical zone (P) and medullary sinuses (MS), (H&E, ×100). **B**. CD79α-immunostaining detects mature B-cells, predominantly located in the follicles, and plasma cells in the paracortical zone (IHC method, ×100). **C**. CD3-immunostaining detects T-cells, predominantly located in the paracortical zone (IHC method, ×100).

**Figure 3:**
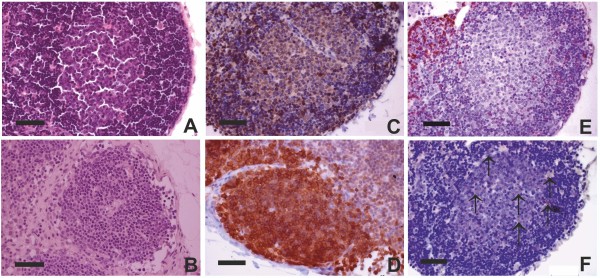
**Follicular hyperplasia**. **A**. Enlarged follicle with a light germinal centre (LAE442, 3 months, H&E, × 400). **B**. Physiological follicle (Control group, H&E, × 400). **C**. B-cells demonstrate a clear expression of CD79α (IHC method, × 4 00). **D**. B cells in a physiological follicle (IHC method, × 400). **E**. CD3 + T-cells presented in follicles (LAE442, 3 months, IHC method, × 400). **F**. Single CD68+ macrophages presented in follicles (arrows) (LAE442, 3 months, IHC method, × 400).

The control groups with surgery but without implant showed the presence of sinus histiocytosis, follicular hyperplasia and heterophilic infiltration with low score values overall and demonstrated decrease of all investigated characteristic values from three to six months. The control group without surgery had the lowest score values in all investigated parameters. Expression of CD3 and CD79α in physiological (Figure 2B, 2C) as well as in affected lymph nodes was conformed to T- and B-dependent zones (Figure 3C, 3E). CD3 was expressed in T-cells of the paracortical zone as clear membrane staining. Single T-cells were found in cortex near or within the follicles. There were no significant differences for CD3 expression in all groups and times including the control group without surgery (Figure 2C). CD79α was expressed in B-cells of the cortex, primary (Figure 2B) and secondary follicles, and rarely in medullary cords (plasma cell reaction). A few of B-cells and plasma cells were found in sinuses.

### Sinus histiocytosis

**Figure 4:**
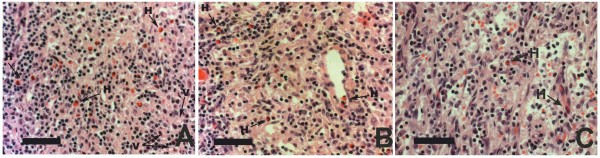
**Sinus histiocytosis**. **A**. Histiocytes in the central sinuses of the lymph node after six months exposure to MgCa0.8 (H&E, × 400). Note the histiocytes with non-stained vacuoles (V) and the diffuse heterophilic infiltration (H). **B**. H&E staining after six months exposure to TiAl6V4 (H&E, × 400). Heterophilic infiltration is present (H) but no vacuoles could be observed. **C**. Physiological lymph node (control group without surgery, H&E staining, × 400). Black bar = 50 μm.

**Figure 5:**
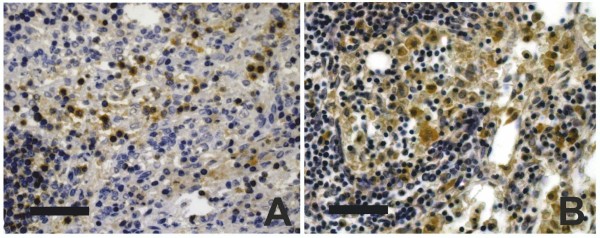
**Sinus histiocytosis**. **A**. Histiocytes in the central sinuses of the lymph node after three months exposure to MgCa0.8 and **B**. after three months exposure to TiAl6V4 (CD68-immunostaining of histiocytes, × 400). Sinus histiocytosis was increased in lymph nodes of the TiAl6V4-group in comparison to the MgCa0.8-group. Black bar = 50 μm.

**Figure 6:**
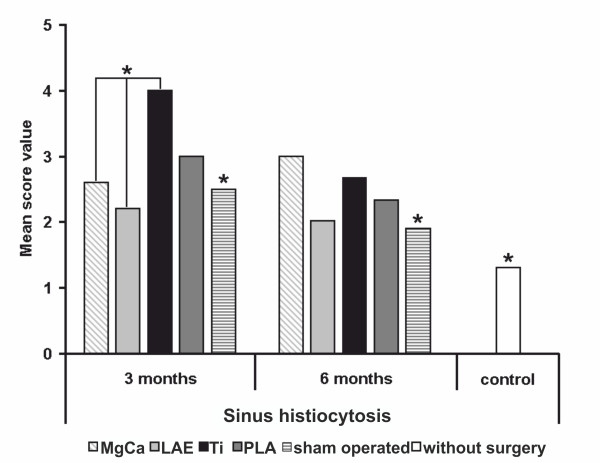
Score values of sinus histiocytosis for all examined groups (* = *p *< 0.05). After three months observation period, sinus histiocytosis was significantly increased in the Ti-group in comparison to the groups with the magnesium based alloys LAE and MgCa. MgCa = MgCa0.8, LAE = LAE442, PLA = poly-96L/4D-lactide, Ti = TiAl6V4, sham operated = surgery without implant material, control = no surgery on the limb.

The most widespread change in all groups was sinus histiocytosis except the control group without surgery which showed a low score value (SV 1.3). This reactive pattern is characterized by distension and prominence of the lymphatic sinusoids, due to a marked hypertrophy of lining endothelial cells and an infiltration of histiocytes. Numerous histiocytes in lymph nodes from both groups with magnesium-based implants (MgCa0.8 and LAE442) were found with foamy cytoplasm and non-stained vacuoles which contained small pale basophilic particles (Figure 4A). Histiocytes were recognized as CD68-positive cells (Figure 5) and were located in central and marginal sinuses, though single cells occurred within the cortical and paracortical zones (Figure 3F). After three months implantation duration, the titanium group showed the highest score values for sinus histiocytosis (score value 4), which were significant higher than both magnesium based alloys, the sham operated group and the control without surgery (MgCa0.8 SV 2.6, LAE442 SV 2.2, sham operated SV 2.5, control without surgery SV 1.3, p < 0.05). The PLA-group demonstrated a moderate level of this parameter (SV 3.0). While the score values for titanium and PLA decreased from three to six months (Ti, SV 2.7 and PLA, SV 2.3), the value for MgCa0.8 increased insignificantly (SV 3.0) so that it exceeded those of PLA and titanium. LAE442 showed the lowest score values of all groups with implant material after both implantation periods with a few tendency of decrease over the time (SV 2.2 and 2.0), very similar to the sham operated group (SV 2.5 and 1.9) (Figure 6).

### Apoptosis of histiocytes

**Figure 7:**
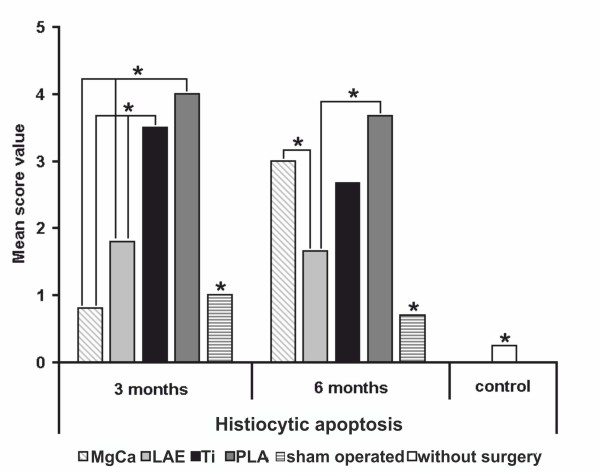
Score values of histiocytic apoptosis for all examined groups (* = *p *< 0.05). Three months: PLA and Ti showed significant higher values than MgCa, LAE, sham operated and control group; Six months: MgCa showed a distinct increase, the values for PLA, Ti, LAE and for the sham operated group decreased. MgCa = MgCa0.8, LAE = LAE442, PLA = poly-96L/4D-lactide, Ti = TiAl6V4, sham operated = surgery without implant material, control = no surgery on the limb.

The most distinct differences between the material groups could be found with the evaluation of histiocytic apoptosis. After three months implantation duration PLA (SV 4.0) and titanium (SV 3.5) showed significant higher values (p < 0.05) than MgCa0.8 (score 0.8), with the lowest level of apoptosis, LAE442 (score 1.8) and both control groups (sham operated SV 1.0 and control without surgery SV 0.3). After six months of implantation duration the results changed obviously. MgCa0.8 showed a distinct increase in the number of apoptotic histiocytes (score value 3.0, p < 0.05). Contrary, the values for PLA (SV 3.7), titanium (SV 2.7), LAE442 (SV 1.6) and for the sham operated group (SV 0.7) decreased. LAE442 showed clearly lower score values than all other material groups (Figure 7).

### Heterophilic infiltration

**Figure 8:**
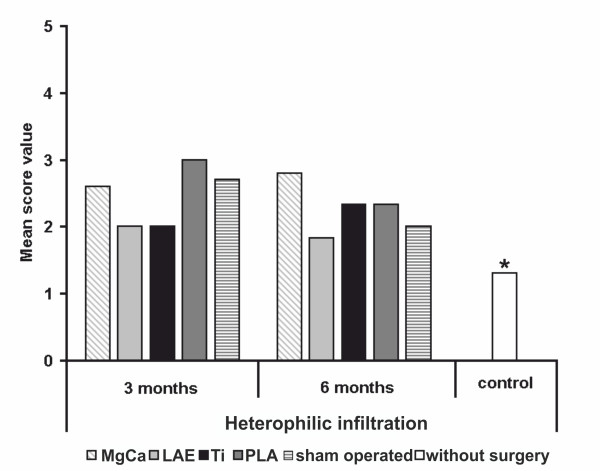
**Score values of heterophilic infiltration for all examined groups**. Note the absence of significant differences between all groups and evaluation times, except for the control group without surgery (* = *p *< 0.05). MgCa = MgCa0.8, LAE = LAE442, PLA = poly-96L/4D-lactide, Ti = TiAl6V4, sham operated = surgery without implant material, control = no surgery on the limb.

Heterophils were found in sinuses (Figure 4) and, rarely, in follicles and the paracortical zone. After three months of implantation duration, MgCa0.8, PLA and the sham operated group showed higher score values for heterophilic infiltration (SV 2.6, SV 3.0 and SV 2.7) than LAE442 and titanium (both SV 2.0) as well as the control group without surgery (SV 1.3). While the score increased for MgCa0.8 (SV 2.8) and thus showed the highest values of all materials after six months the score value for PLA decreased (SV 2.3) and reached a similar level as titanium (SV 2.3). LAE442 (SV 1.8) showed the lowest level of all tested materials, even lower than the sham operated group (SV 2.0) after six months implantation duration. Described differences were not statistically significant between the investigated groups (Figure 8). However, they were significantly higher than in the control group without surgery.

### Follicular hyperplasia

**Figure 9:**
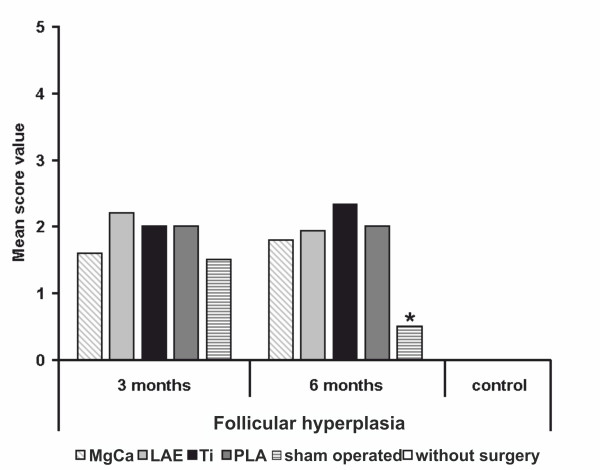
**Score values of follicular hyperplasia for all examined groups**. Note the absence of significant differences between all groups and evaluation times, except for the sham operated group after six months and the control group without surgery (* = *p *< 0.05). MgCa = MgCa0.8, LAE = LAE442, PLA = poly-96L/4D-lactide, Ti = TiAl6V4, sham operated = surgery without implant material, control = no surgery on the limb.

The lymph nodes from the control group without surgery did not show any signs of follicular hyperplasia (SV 0.0). After three months implantation duration, follicular hyperplasia was found in a moderate level for all material groups (LAE442, SV 2.2; PLA and Ti, SV 2.0; MgCa0.8, SV 1.6) as well as in the sham operated group (SV 1.5). After six months, a significant decrease could only be seen in the control group without surgery (SV 0.5), a slight decrease was found in LAE442 (SV 1.9). The other material groups showed no difference over time (PLA, SV 2.0) or a slight increase (MgCa0.8, SV 1.8) (Figure 9). The increase of CD79α expression was corresponded to the presence of follicular hyperplasia in H&E-staining (Figure 3). However, as was mentioned above, there were no meaningful differences between all groups and times.

## Discussion

The presented study was performed to demonstrate and compare the morphological changes in peripheral lymphoid tissue, which can reflect the condition of local immunity after the exposure to commonly used (PLA) and newly developed (MgCa0.8 and LAE442) resorbable and non-resorbable (titanium) bone implants in comparison to control groups. The results show, that the four examined implant materials as well as the surgical procedure itself provoke similar reactions within the regional lymphoid tissue, which were sinus histiocytosis, follicular hyperplasia, heterophilic infiltration and apoptosis of histiocytes. The changes in efferent lymph nodes in all material groups after observation periods of three and six months were mainly moderate without significant differences in comparison to the control groups with surgery for heterophilic infiltration and follicular hyperplasia. Higher than moderate values were only demonstrated in the commonly used materials titanium, for sinus histiocytosis and apoptosis of histiocytes (titanium, 3 months), and PLA, for sinus histiocytosis (PLA, 3 and 6 months). Lee et al. investigated the release of metal elements from titanium implants [40]. Titanium particles were neither detected in periimplant soft tissue nor in regional lymph nodes. However, it cannot be excluded that titanium implants induce an immuno-inflammatory response leading to reactions in regional lymph nodes.

The presence of sinus histiocytosis, follicular hyperplasia and heterophilic infiltration in the examined lymph nodes could be explained by different but joined ways of immune reaction. Follicular hyperplasia develops in lymph nodes which react to inflammatory processes in the area they filter. The offending antigen is brought into the lymph node by lymphatic drainage, where it is phagocytized and degraded within macrophages, and therewith initiates an immune reaction by activating B-cells, which enter into B-cell follicles and create the reaction of a germinal centre [33]. The rare occurrence of follicular hyperplasia, its relatively low range and absence of significant differences between the control groups without implant and investigated material groups allow the conclusion of an absence of antigen features in all investigated implant materials. Moreover, the visual differences between the groups for the parameters sinus histiocytosis and heterophilic infiltration lead to the hypothesis, that the immune response to the investigated implants predominantly is a foreign-body reaction [5], which is substantiated in our study by the development of a chronic non-specific lymphadenitis. No significant differences for CD3 expression between all groups and times may also indicate the non-specific character of immune response to investigated materials. In consideration of the fact that heterophils or neutrophils as well as histiocytes take part in scavenging degradation microparticles [33], the presence of non-stained intracellular inclusions in abundant histiocytes in our study seem to be obvious reactions. Similar intracellular inclusions were also reported in other studies [5,31,41].

As described in previous literature, titanium implants used in the treatment of fractures caused an immuno-inflammatory reaction in adjacent soft tissue [42,43]. These studies showed that a marked inflammation was present in the soft tissue covering the titanium implants [42] and that titanium particles were included in the cytoplasm of macrophages [43]. Furthermore, the oxidation products of titanium, especially titanium dioxide, can cause increase of apoptosis in vivo [19] and in vitro [20]. Meng et al. reported the cytotoxic effect of titanium microparticles on the bone marrow stem cells adjacent to the titanium implant due to phagocytosis of the particles by these cells, following the increased expression of tumor suppressor protein p53 [23]. Phagocytized microparticles of PLA also can induce apoptosis of macrophages in vivo [22]. Recent studies described the short-term (24 h) concentration-depended apoptotic-stimulating effects of Mg^2+-^- and Ca^2+^- ions as well as some rare-earth metals on cell lines [28]. However no data about the long-term apoptotic effects have been found in available literature. Dribben et al. reported on the increase of apoptosis in fetal mouse neurons under the influence of high-concentrated magnesium-ions [29]. Evidently, appearance of macrophage apoptosis could be induced by external agents, like products of degradation and oxidation of implants [19,20]. Therefore, the same properties could be suggested for the magnesium alloys examined in this study, as well as for the other materials. This thesis is also supported by a study of Sun and Cohen which showed a role of free calcium and magnesium ions in the early apoptotic degradation of DNA by activation of Mg^2+^/Ca^2+^-dependent endonuclease [30]. Theoretically, the fact of Mg^2+^/Ca^2+^-dependent endonuclease activation by free Mg^2+^- and Ca^2+^- ions [30] as well as the fact of p53-level increase by titanium microparticles [23] allow to assume the hypothesis that titanium microparticles, free magnesium- and calcium-ions seem to induce the apoptosis similarly when DNA is injured. DNA damage leads to the accumulation of the p53 protein in cells. It first arrests the cell cycle (at the G_1 _phase) to allow time for repair [33]. However, if the damage is too great to be repaired successfully, p53 triggers apoptosis, mainly by activating sensors that ultimately activate *Bax *and *Bak*, and by stimulating synthesis of pro-apoptotic members of the *Bcl-2 *family [33]. Nonetheless, the exact pathway of apoptosis development in histiocytes in the present study remains unclear and has to be investigated in further studies.

Statistically significant differences between implantation periods could only be found for sinus histiocytosis in the titanium groups and for apoptotic histiocytes in the MgCa0.8-groups. First of all, the sinus histiocytosis level after implantation of titanium implants decreased from three to six months, which may be caused by the decrease of monocytic migration to the implant surface due to progressive ossification and maturation of fibrous tissue around this non-resorbable implant [5].

Due to the ambiguity of exact apoptotic mechanism, the increase of the score value for apoptotic histiocytes in the MgCa0.8-group could not be completely explained. Hypothetically, the high degradation rate of MgCa0.8 [2] and the progressive release of degradation products might induce apoptosis [29,30]. Nevertheless, this suggestion should to be verified carefully in further studies. In experimental studies in rabbits, MgCa- as well as LAE442-alloys are tested with a good in-vivo biocompatibility and are described as possible implant materials for orthopaedic applications [36-38]. With respect to the practical implementation, it is important to note that LAE442 induced the lowest morphological changes with a low immunogenic potential. Hypothetically, these findings are due to its lower degradation rate in comparison to MgCa0.8 implants [2,37,38] and therefore less influence of degradation products on the surrounding tissue.

In consideration of the presented data, it could be concluded that the evaluation of morphological changes in the regional popliteal lymph nodes is useful for the estimation of implant immunocompatibility. Immunohistochemistry was quite helpful for the sinus histiocytosis and follicular hyperplasia evaluation via identification of CD3, CD68 and CD79α expression. Histological and immunohistochemical examination in our study demonstrated the common pathway of immune response for all investigated implants: the development of foreign-body reaction in the local area, followed by the development of chronic, non-specific lymphadenitis in the regional lymph nodes.

## Conclusion

Although immunological reactions to titanium and PLA are observed, these materials were accepted as biocompatible implant materials in clinical use. Immunological reactions to MgCa0.8 increased during the observation period, but did not significantly exceed the reactions to titanium and PLA. LAE442 induced even lower morphological changes. For this reason, LAE442 seems to be a promising candidate as degradable implant material and MgCa0.8 might be acceptable as well. However, further research is required to deepen the understanding of immune response to bone implants and to clarify the pathway of apoptosis, which is induced by the degradation products of different implant materials.

## List of Abbreviations

wt%: weigth percent; LAE442: magnesium alloy with 90 wt% magnesium, 4wt% lithium, 4 wt% aluminium and 2wt% rare earth composition metal; MgCa0.8: magnesium alloy with 99.2 wt% magnesium, 0.8 wt% calcium; SV: score value; MV : mean value.

## Competing interests

The authors declare that they have no competing interests.

## Authors' contributions

AB performed the histological and immunohistochemical examinations, analysed the data and wrote the manuscript. MHT supervised the histological and immunohistochemical examinations. NE participated in the histological and immunohistochemical examinations and helped to draft the manuscript. NA participated in the animal experiment and helped to draft the manuscript. JR participated in the animal experiment and helped to draft the manuscript. AML initiated and conceived of the study, participated in its design and coordination and participated in the animal experiment. All authors read and approved the final manuscript.

## References

[B1] Witte F Kaese V Haferkamp H Switzer E Meyer-Lindenberg A Wirth CJ Windhagen H In vivo corrosion of four magnesium alloys and the associated bone response Biomaterials 2005 26 3557 3563 10.1016/j.biomaterials.2004.09.049 15621246

[B2] Krause A von der Höh N Bormann D Krause C Bach F Windhagen H Meyer-Lindenberg A Degradation behaviour and mechanical properties of magnesium implants in rabbit tibiae J Mater Sci 2010 45 624 632 10.1007/s10853-009-3936-3

[B3] Witte F Abeln I Switzer E Kaese V Meyer-Lindenberg A Windhagen H Evaluation of the skin sensitizing potential of biodegradable magnesium alloys J Biomed Mater Res A 2007 86 1041 1047 10.1002/jbm.a.31713 18067164

[B4] Shive MS Anderson JM Biodegradation and biocompatibility of PLA and PLGA microspheres Adv Drug Deliv Rev 1997 28 5 24 10.1016/S0169-409X(97)00048-3 10837562

[B5] Witte F Ulrich H Rudert M Willbold E Biodegradable magnesium scaffolds: Part 1: appropriate inflammatory response J Biomed Mater Res A 2007 81 748 756 17390368 10.1002/jbm.a.31170

[B6] Thomann M Krause C Bormann D von der Höh N Windhagen H Meyer-Lindenberg A Comparison of the resorbable magnesium alloys LAE442 und MgCa0.8 concerning their mechanical properties, their progress of degradation and the bone-implant-contact after 12 months implantation duration in a rabbit model Mat-wiss u Werkstofftech 2009 40 82 87 10.1002/mawe.200800412

[B7] Peuster M Wohlsein P Brügmann M Ehlerding M Seidler K Fink C Brauer H Fischer A Hausdorf G A novel approach to temporary stenting: degradable cardiovascular stents produced from corrodible metal-results 6-18 months after implantation into New Zealand white rabbits Heart 2001 86 563 569 10.1136/heart.86.5.563 11602554 PMC1729971

[B8] Quadbeck P Hauser R Kümmel K Standke G Stephani G Nies B Rößler S Wegener B Iron Based Cellular Metals For Degradable Synthetic Bone Replacement 2010 PM2010 World Congress, Florenz, Italy

[B9] Hallab N Jacobs J Black J Hypersensitivity associated with metallic biomaterials Biomater Eng and Devices 2000 1 1 7

[B10] Merritt K Rodrigo JJ Immune response to synthetic materials. Sensitization of patients receiving orthopaedic implants Clin Orthop Relat Res 1996 326 71 79 8620661

[B11] Griem P Gleichmann E Metal ion induced autoimmunity Current Opinion in Immunology 1995 7 831 838 10.1016/0952-7915(95)80056-5 8679128

[B12] Griem P von Vultée C Panthel K Best SL Sadler PJ Shaw CF T cell cross-reactivity to heavy metals: identical cryptic peptides may be presented from protein exposed to different metals Eur J Immunol 1998 28 1941 1947 10.1002/(SICI)1521-4141(199806)28:06<1941::AID-IMMU1941>3.0.CO;2-H 9645376

[B13] Kubicka-Muranyi M Griem P Lübben B Rottman N Lührmann R Beyer KG Mercuric chloride-induced autoimmunity in mice involves an upregulated presentation of altered and unaltered nucleolar self antigen Int Arch Allergy Immunol 1995 108 1 10 7647579 10.1159/000237110

[B14] Yang J Merritt K Production of monoclonal antibodies to study corrosion products of CO-CR biomaterials J Biomed Mater Res 1996 31 71 80 10.1002/(SICI)1097-4636(199605)31:1<71::AID-JBM9>3.0.CO;2-N 8731151

[B15] Gil-Albarova J Laclériga A Barrios C Cañadell J Lymphocyte response to polymethylmethacrylate in loose total hip prostheses J Bone Joint Surg Br 1992 74 825 830 1447242 10.1302/0301-620X.74B6.1447242

[B16] Angle CR Organ-specific therapeutic intervention Metal toxicology 1995 Goyer RA, Klaasen CD. San Diego: Academic Press 71 110

[B17] Lalor PA Revell PA Gray AB Wright S Railton GT Freeman MA Sensitivity to titanium. A cause of implant failure? J Bone Joint Surg Br 1991 73 25 28 1991768 10.1302/0301-620X.73B1.1991768

[B18] Parker AW Drez D Jr Jacobs JJ Titanium dermatitis after failure of a metal-backed patellas Am J Knee Surg 1993 6 129 131

[B19] Wang L Mao J Zhang G Tu M Nano-cerium-element-doped titanium dioxide induces apoptosis of Bel 7402 human hepatoma cells in the presence of visible light World J Gastroenterol 2007 13 4011 4014 17663520 10.3748/wjg.v13.i29.4011 PMC4171178

[B20] Rahman Q Lohani M Dopp E Pemsel H Jonas L Weiss DG Schiffmann D Evidence that ultrafine titanium dioxide induces micronuclei and apoptosis in Syrian hamster embryo fibroblasts Environ Health Perspect 2002 110 797 800 10.1289/ehp.02110797 12153761 PMC1240951

[B21] Bursch W Taper HS Lauer B Schulte-Hermann R Quantitative histological and histochemical studies on the occurrence and stages of controlled cell death (apoptosis) during regression of rat liver hyperplasia Virchows Arch, B, Cell Pathol 1985 50 153 166 2868562 10.1007/BF02889898

[B22] Lam KH Schakenraad JM Esselbrugge H Feijen J Nieuwenhuis P The effect of phagocytosis of poly(L-lactic acid) fragments on cellular morphology and viability J Biomed Mater Res 1993 27 1569 1577 10.1002/jbm.820271214 8113245

[B23] Meng B Chen J Guo D Ye Q Liang X The effect of titanium particles on rat bone marrow stem cells in vitro Toxicol Mech Methods 2009 19 552 558 10.3109/15376510903401716 19874181

[B24] Hori N Att W Ueno T Sato N Yamada M Saruwatari L Suzuki T Ogawa T Age-dependent degradation of the protein adsorption capacity of titanium J Dent Res 2009 88 663 667 10.1177/0022034509339567 19641155

[B25] Black J Systemic effects of biomaterials Biomaterials 1984 5 11 18 10.1016/0142-9612(84)90061-9 6375744

[B26] Jacobs JJ Corrosion of metallic implants Advances in operative orthopaedics 1994 1 Stauffer, RN. St. Louis: CV Mosby 279 319

[B27] Yang J Black J Competitive binding of chromium, cobalt and nickel to serum proteins Biomaterials 1994 15 262 268 10.1016/0142-9612(94)90049-3 8031985

[B28] Feyerabend F Fischer J Holtz J Witte F Willumeit R Drücker H Vogt C Hort N Evaluation of short-term effects of rare earth and other elements used in magnesium alloys on primary cells and cell lines Acta Biomater 2010 6 1834 1842 10.1016/j.actbio.2009.09.024 19800429

[B29] Dribben WH Creeley CE Wang HH Smith DJ Farber NB Olney JW High dose magnesium sulfate exposure induces apoptotic cell death in the developing neonatal mouse brain Neonatology 2009 96 23 32 10.1159/000201327 19204407 PMC3087884

[B30] Sun XM Cohen GM Mg(2+)-dependent cleavage of DNA into kilobase pair fragments is responsible for the initial degradation of DNA in apoptosis J Biol Chem 1994 269 14857 14860 8195114

[B31] Verheyen CC de WR van BA Rozing PM de Groot K Examination of efferent lymph nodes after 2 years of transcortical implantation of poly(L-lactide) containing plugs: a case report J Biomed Mater Res 1993 27 1115 1118 10.1002/jbm.820270817 8408124

[B32] Ferreira ME de Lourdes Pereira M Garcia e Costa F Sousa JP de Carvalho GS Comparative study of metallic biomaterials toxicity: a histochemical and immunohistochemical demonstration in mouse spleen J Trace Elem Med Biol 2003 17 45 49 10.1016/S0946-672X(03)80045-7 12755501

[B33] Kumar V Abbas AK Fausto N Robbins and Cotran pathologic basis of disease 2004 Elsevier/Saunders

[B34] Albores-Saavedra JM Vuitch FM Delgado RM Wiley EM Hagler H Sinus Histiocytosis of Pelvic Lymph Nodes after Hip Replacement: A Histiocytic Proliferation Induced by Cobalt-Chromium and Titanium Am J Surg Pathol 1994 18 83 90 10.1097/00000478-199401000-00008 8279630

[B35] Munichor M Cohen H Volpin G Kerner H Iancu TC Chromium-induced lymph node histiocytic proliferation after hip replacement. A case report Acta Cytol 2003 47 270 274 10.1159/000326515 12685200

[B36] Erdmann N Bondarenko A Hewicker-Trautwein M Angrisani N Reifenrath J Lucas A Meyer-Lindenberg A Evaluation of the soft tissue biocompatibility of MgCa0.8 and surgical steel 316L in vivo: a comparative study in rabbits Biomed Eng Online 2010 9 63 10.1186/1475-925X-9-63 20974008 PMC2976742

[B37] Reifenrath J Krause A Bormann D von Rechenberg B Windhagen H Meyer-Lindenberg A Profound differences in the in-vivo-degradation and biocompatibility of two very similar rare-earth containing Mg-alloys in a rabbit model Mat-wiss u Werkstofftech 2010 41 1054 1061 10.1002/mawe.201000709

[B38] Li Z Gu X Lou S Zheng Y The development of binary Mg-Ca alloys for use as biodegradable materials within bone Biomaterials 2008 29 1329 1344 10.1016/j.biomaterials.2007.12.021 18191191

[B39] Jones M Cordell JL Beyers AD Tse AG Mason DY Detection of T and B cells in many animal species using cross-reactive anti-peptide antibodies J Immunol 1993 150 5429 5435 8515069

[B40] Lee S Goh BT Lai SH Tideman H Stoelinga PJ Jansen JA Peri-implant and systemic release of metallic elements following insertion of a mandibular modular endoprosthesis in Macaca fascicularis Acta Biomater 2009 5 3640 3646 10.1016/j.actbio.2009.05.028 19481181

[B41] Krause A Untersuchung der Degradation und Biokompatibilität von degradablen, intramedullären Implantaten auf Magnesiumbasis im Kaninchenmodell Dissertation 2008 Hannover: Tierärztliche Hochschule

[B42] Voggenreiter G Leiting S Brauer H Leiting P Majetschak M Bardenheuer M Obertacke U Immuno-inflammatory tissue reaction to stainless-steel and titanium plates used for internal fixation of long bones Biomaterials 2003 24 247 254 10.1016/S0142-9612(02)00312-5 12419625

[B43] Katou F Andoh N Motegi K Nagura H Immuno-inflammatory responses in the tissue adjacent to titanium miniplates used in the treatment of mandibular fractures J Craniomaxillofac Surg 1996 24 155 162 8842906 10.1016/s1010-5182(96)80049-7

